# Supplementary material for: Modeling Gastrointestinal Tract Wet Pool Size in Small Ruminants
Source: Animals (Basel). 2023 Sep 13;13(18):2909. doi: 10.3390/ani13182909 (PMC10525868; doi:10.3390/ani13182909)
Supplement: Supplementary file 1 [file animals-13-02909-s001.zip › animals-2500697-supplementary.pdf]

animals

Supplementary Material

Modeling gastrointestinal tract wet pool size in small ruminants

Paola R. Ribeiro[1], Marcelo Gindri[2], Gilberto L. Macedo Junior[3], Caio J. L. Herbster[4], Elzania S. Pereira[4], Bruno Biagioli[1], and Izabelle A. M. A. Teixeira[1,5,*]

[1] Department of Animal Science, São Paulo State University, Jaboticabal 14884900, SP, Brazil; paola.rezenderibeiro@gmail.com (P.R.R.); bruno.biagioli@unesp.br (B.B.)
[2] UMR Modélisation Systémique Appliquée aux Ruminants, AgroParisTech, INRAE,Université Paris-Saclay, 91120, Palaiseau, France; gindri.marcelo@gmail.com
[3] Department of Animal Science, Federal University of Uberlândia, Uberlândia 38402018, MG, Brazil; gilberto.macedo@ufu.br
[4] Department of Animal Science, Federal University of Ceará, Fortaleza 60356000, CE, Brazil; julioherbster@gmail.com (C.J.L.H.); elzania@hotmail.com (E.S.P.)
[5] Department of Animal Veterinary, and Food Sciences, University of Idaho, Twin Falls, ID 83301, USA
* Correspondence: izabelle@uidaho.edu

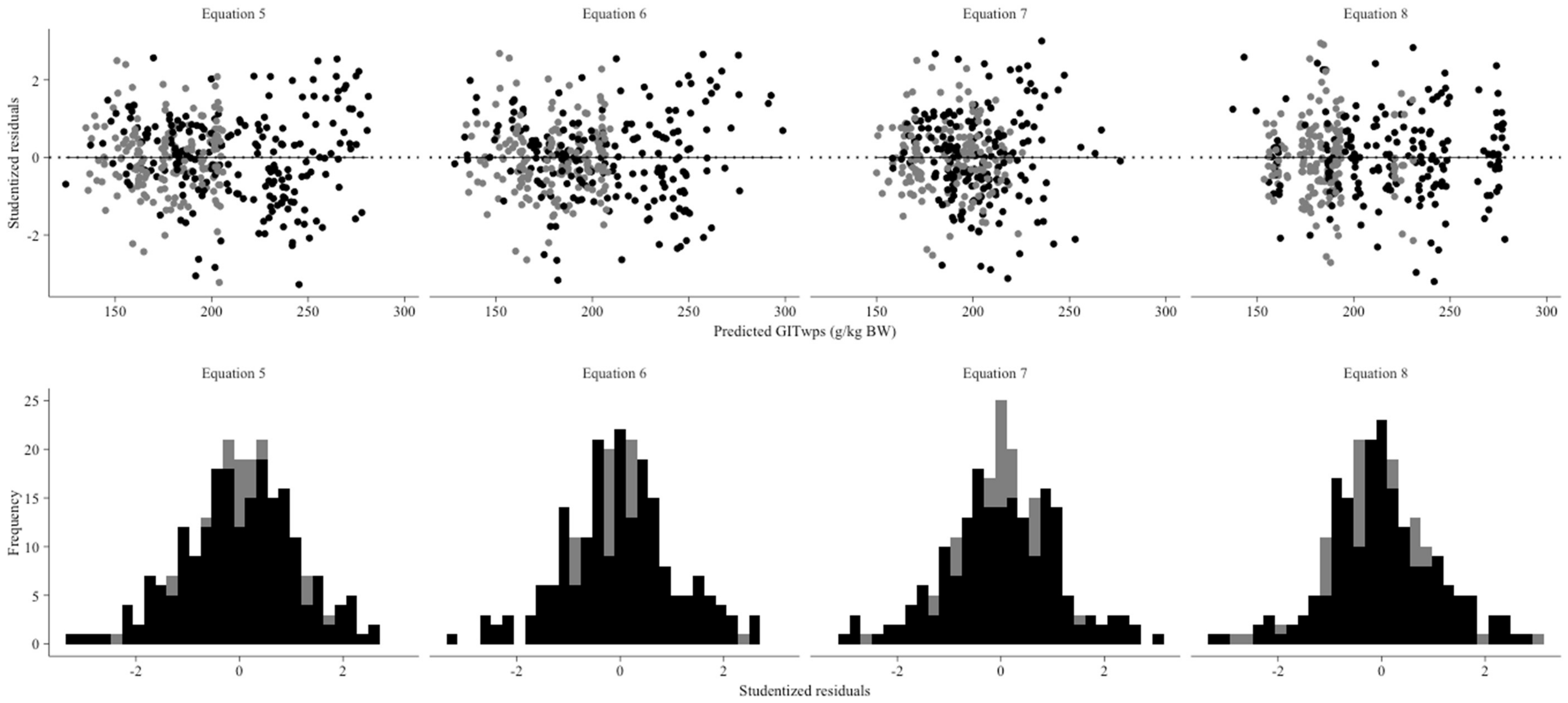

Figure S1: Studentized residuals of the linear equations proposed to describe the gastrointestinal tract wet pool size (GITwps) of small ruminants. Goats are grey and sheep are black.

animals

MDPI

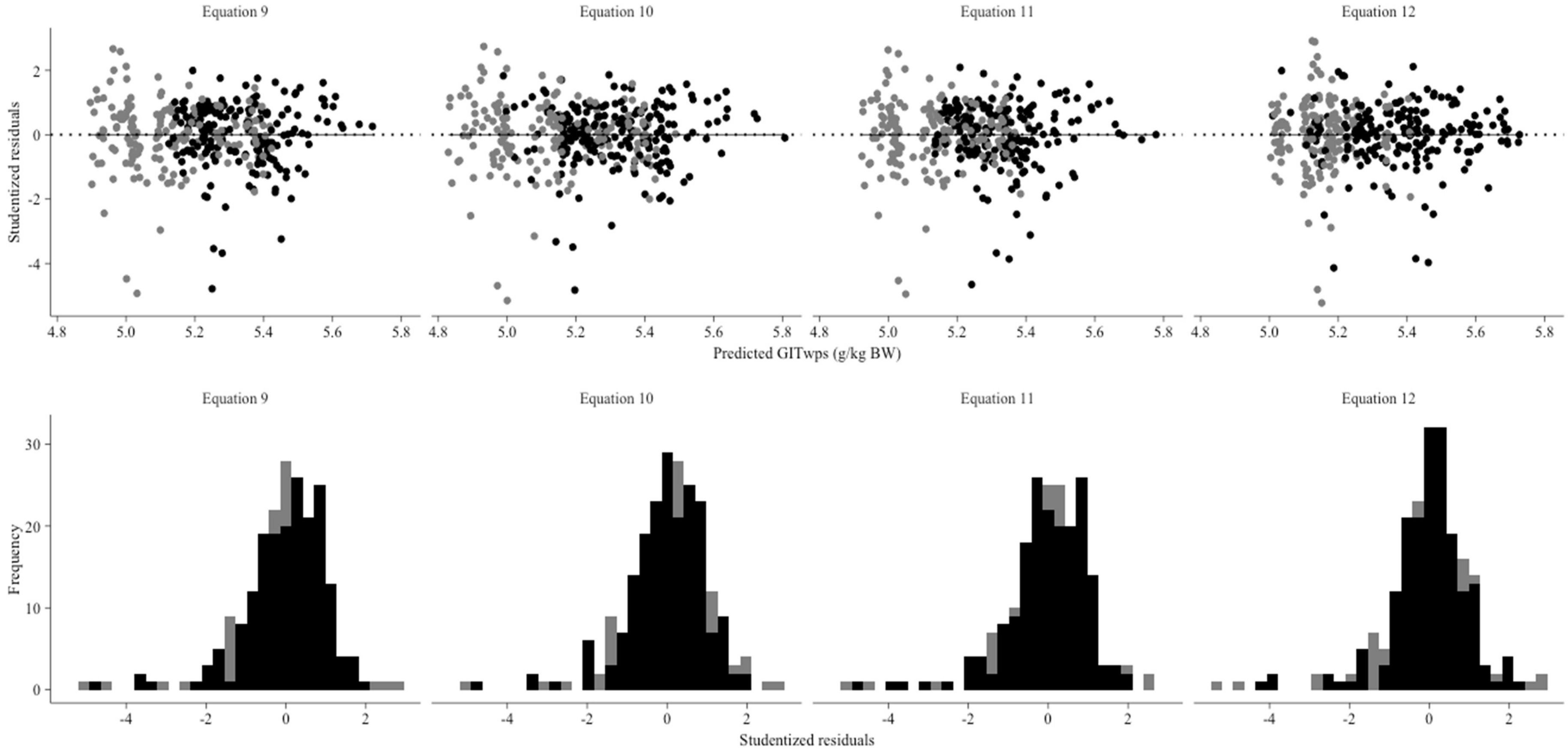

Figure S2: Studentized residuals of the non-linear equations proposed to describe gastrointestinal tract wet pool size (GITwps) of small ruminants. Goats are grey and sheep are black.
